# Applying novel connectivity networks to wood turtle populations to provide comprehensive conservation management strategies for species at risk

**DOI:** 10.1371/journal.pone.0271797

**Published:** 2022-08-12

**Authors:** Cindy Bouchard, Étienne Lord, Nathalie Tessier, François-Joseph Lapointe

**Affiliations:** 1 Département de sciences biologiques, Université de Montréal, Montréal, Québec, Canada; 2 Ministère des Forêts, de la Faune et des Parcs, Longueuil, Québec, Canada; USDA Forest Service, UNITED STATES

## Abstract

Genetic diversity within and among populations is frequently used in prioritization processes to rank populations based on their vulnerability or distinctiveness, however, connectivity and gene flow are rarely considered within these frameworks. Using a wood turtle (*Glyptemys insculpta*) population graph, we introduce BRIDES as a new tool to evaluate populations for conservation purpose without focusing solely on individual nodes. BRIDES characterizes different types of shortest paths among the nodes of a subgraph and compares the shortest paths among the same nodes in a complete network. The main objectives of this study were to (1) introduce a BRIDES selection process to assist conservation biologists in the prioritization of populations, and (2) use different centrality indices and node removal statistics to compare BRIDES results and assess gene flow among wood turtle populations. We constructed six population subgraphs and used a stepwise selection algorithm to choose the optimal number of additional nodes, representing different populations, required to maximize network connectivity under different weighting schemes. Our results demonstrate the robustness of the BRIDES selection process for a given scenario, while inconsistencies were observed among node-based metrics. Results showed repeated selection of certain wood turtle populations, which could have not been predicted following only genetic diversity and distinctiveness estimation, node-based metrics and node removal analysis. Contrary to centrality measures focusing on static networks, BRIDES allowed for the analysis of evolving networks. To our knowledge, this study is the first to apply graph theory for turtle conservation genetics. We show that population graphs can reveal complex gene flow dynamics and population resiliency to local extinction. As such, BRIDES offers an interesting complement to node-based metrics and node removal to better understand the global processes at play when addressing population prioritization frameworks.

## Introduction

The conservation of units below the species level, commonly populations, is widely used in wildlife management, as it provides critical information to support species survival and evidence of within-species differences. Populations show important local adaptive variations that are essential for species adaptation to changing environments [1–3]. Furthermore, they exhibit greater sensitivity to extirpation, which may inform of the general trend at the species level [4]. The threats experienced by animal and plant populations require distinct management practices that must consider the economic, social and cultural aspects of the human society living in closest proximity to them [5–7]. Rule-based methods have been developed by the International Union for Conservation of Nature (IUCN) to categorize populations based on the level of threat they face [8–11]. Such multiple criteria have been used to categorize species at risk and aid governmental and non-governmental organizations to define conservation units, including: Management Units (MUs, [2, 12]), Evolutionary Significant Units (ESUs, [2]) and Designable Units (DUs). The latter is used by the Committee on the Status of Endangered Wildlife in Canada (COSEWIC) to define populations or subspecies of wildlife that require protection under the Species at Risk Act (SC 2002, c 29).

Considering that two populations may be ranked in the same category even when faced with entirely different threats, and the limited financial resources available for conservation biology, prioritizing populations arose as an essential concept to target the most effective conservation actions. Multiple prioritization frameworks have been developed to provide objective criteria during decision-making processes, and either estimate species extinction risks [5] or prioritize threatened populations and determine the management actions required to support them [13]. Prioritization frameworks are often reactive, targeting highly vulnerable populations in need of immediate management actions to avoid reducing genetic diversity and local or global species extinction events [14]. However, for species with long generation times, it may be beneficial to react in a proactive fashion and prioritize populations that exhibit low vulnerability to protect their global genetic diversity as a reservoir [15–17].

Population viability analysis (PVA) is frequently used to evaluate extinction risks given its comprehensive and quantitative basis [18]. To complement such models, or, in cases where data are unsuitable, the relative importance of each population can also be estimated to measure the impact of local extinction on species survival. A series of binary questions have been proposed by Allendorf and colleagues [19] for the Pacific salmon (*Oncorhynchus spp*.) and adapted for all freshwater fish by Clarkson and colleagues [20], thus providing a scoring system for the biological consequence of local population extinctions for the entire species. Although these rankings are intended to represent objective criteria, the list of questions may overlook important features that deserve conservation attention.

Measures of genetic diversity within and among populations are frequently applied to prioritize populations and define conservation units based on past bottleneck events, effective population size and population distinctiveness [21, 22]. To bridge the gap between researchers and practitioners, Ottewell and colleagues [22] developed a simple framework using population differentiation, genetic diversity, and inbreeding coefficients. The quantification and ranking of these parameters can be of great importance to estimate population isolation, predict their trends and target the most informative genetic processes for management purposes. Although many of these estimators allow to understand the distinctiveness and diversity of a population to predict its persistence, only few consider the dynamic structure underlying population networks. Some demographic studies have accounted for population connectivity by using a metapopulation model in their prioritization framework. However, the data requirements of complex metapopulation models greatly limit their application in conservation studies, calling for a short-term decision-making time frame. For example, the incidence function model of metapopulation dynamics [23] requires estimates of patch colonization and extinction rates that are obtained through longitudinal surveys of all available habitat patches [24]. Yet, connectivity among isolated habitat patches is essential to several ecological processes and should be considered in population prioritization models. Seasonal migration, dispersal of offspring, recolonization following local extinction events, range shifts caused by climate change, and gene flow for the transmission of favourable alleles may all be distorted if change occurs within the connectivity pattern [7, 25].

Gene flow is frequently cited as an evolutionary force that maintains connectivity among populations [26, 27], but connectivity, in itself, can potentially operate at different time scales other than demographic processes, such as dispersion and recolonization events [28, 29]. As such, gene flow should also be considered in population prioritization as it offers complementary insights into population connectivity [28, 30]. Theoretical and empirical studies support the use of gene flow as a measure of functional connectivity [29, 31–33], and graph theory is a valuable tool emphasizing the relevance of connectivity in conservation planning [34]. The processes underlying the structure of complex networks have been efficiently characterized in many disciplines [35] and are increasingly applied in ecology and conservation where food webs, metapopulation dynamics or reserve networks can be modelled [36]. More precisely, population graphs have been developed by Dyer and Nason [37] as a network depicting populations (the nodes of the graph) connected by their genetic covariance (the edges of the graph). Gene flow has since been modelled for many plant and animal species in marine, freshwater, and terrestrial ecosystems using networks [38–40]. For management purposes, population prioritization can be facilitated by the ranking of several node-based centrality indices [41], or by using node removal to assess their relative impact on network connectivity [29]. Population prioritization has also been studied with phylogenetic networks [42], for which genetic distinctiveness was used to score each population. Yet, the choice of relevant network metrics is crucial for the prioritization process using such methods [43–45].

The wood turtle (*Glyptemys insculpta*) is a threatened freshwater turtle species endemic to North America and listed as endangered according to the IUCN red list, following an overall decrease in population size [46]. It is a semi-terrestrial, long-lived species with longevity that may exceed 50 years [47], delayed sexual maturity at 11–22 years [48, 49], and a long generation time estimated at 36–47 years [46]. Canadian wood turtle populations are protected under the Species at Risk Act, but financial support is lacking to ensure the persistence of all populations. In cases where wildlife managers must decide which populations to protect, a sound prioritization framework is required. Yet, considering the cost and short duration of radiotelemetry studies, the temporal and spatial variation in nest predation, and the difficulty of monitoring hatchlings and juveniles, PVA is not easily applicable to wood turtle populations [50]. Population genetics have been previously used to characterize the population structure of wood turtles [51–54] and the importance of peripheral populations for genetic diversity [55]. In addition, landscape genetics has revealed the importance of the watershed structure for wood turtle population differentiation in Canada [56]. None of these previous studies, however, have relied on graph theory as a statistical tool to analyze connectivity and prioritize populations.

Although many studies highlight the importance of connectivity and gene flow for the maintenance of diversity and the persistence of populations in a fragmented landscape, few frameworks consider connectivity as an important feature of the prioritization ranking. In this paper, we apply BRIDES [57] as a new tool to evaluate whole population graphs for conservation purposes, rather than focusing only on individual patches. Thus, the main objectives of our study are (1) to demonstrate the use of the BRIDES selection algorithm to assist conservation biologists and managers during decision-making processes, and (2) to use graph theory to assess gene flow among wood turtle populations. To do this, we used a dataset of 19 wood turtle populations collected in eastern Canada and genotyped by Bouchard and colleagues [56]. Centrality indices and node removal statistics were first estimated from a population graph. We then constructed six population subgraphs based on three different criteria to protect populations in a proactive or reactive fashion, and using BRIDES, selected the optimal number of additional nodes required to maximize network connectivity under different weighting schemes. The results of the three methods were then applied within the perspective of wood turtle conservation.

## Materials and methods

The sampling protocol was approved by the Ministère des Forêts, de la Faune et des Parcs du Québec.

### Wood turtle population characterization

The original data set from Bouchard and colleagues [56] included 24 sites, but five sites were excluded due to insufficient sample size (N<6). As a result, 327 wood turtles sampled at 19 different sites and genotyped for nine microsatellite loci as described in Bouchard and colleagues [56] were used in the present study. Using microsatellite data, expected heterozygosity (H_E_) was estimated using the diveRsity package with R version 3.5.1 [58]. Element occurrence (EO) ranks were used to estimate the persistence probability of a population for a defined period of time (20–100 years), while considering that current environmental conditions prevailed during that time [59]. These EO ranks vary from excellent viability (A) to poor viability (D), and some rank combinations can be used in cases of uncertain estimates. All EO ranks and estimated population sizes were provided by wildlife biologists at the Québec Ministry of Forests, Wildlife and Parks. Although other measures could have been used to demonstrate the application of population graphs, H_E_ and EO ranks were selected for their frequent use in conservation studies.

### Wood turtle population graph

We constructed a population graph based on Dyer and Nason’s [37] conditional genetic distance (cGD) and pruning method using R v. 3.5.1 [60] popgraph [61] and gstudio [62] packages. In this network, nodes representing different sample sites are connected to each other by undirected edges weighted by the genetic distances between them. However, as some edges do not adequately describe overall among-population distances, the network was pruned by removing redundant edges that did not contribute to the overall genetic covariance of the network, as described by Dyer and Nason [37].

### Node-based metrics of connectivity and node removal

To evaluate the importance of each node with respect to network connectivity, we used two current methods: node-based metrics [45, 63] and the node removal approach [38, 64]. Several node metrics were computed to estimate their contribution to gene flow and importance in graph connectivity. Degree centrality (DC) is defined as the number of edges connected to a node, whereas betweenness centrality (BC) is the number of shortest paths upon which the node lies. Based on immediate neighbour connections, the eigenvector centrality (EC) may also provide information on both direct and indirect levels of node connectivity. The clustering coefficient (CC) is the probability that two nodes connected to a neighboring node are also connected together. Computing strength as the sum of all edge weights connected to a given node is another centrality measure of interest for conservation biology, since the average inverse edge weight (AIEW) is positively correlated with the number of migrants to and from a focal node [63].

The node removal approach was also used to estimate the effect of local extirpation on the connectivity of the network and to obtain an overview of the resilience of the network to node loss [38]. In this study, we simulated the effect of local extirpation by removing a node and its associated edges from the graph and measuring the average path length in the resulting graph. The process was repeated iteratively by removing each node, one at a time. Differences in average path lengths were estimated by comparing the reduced and complete networks, with larger differences being associated with more important nodes for network connectivity. A cut node is a node whose deletion, along with incident edges, results in a disconnected graph. Node-based metrics and node removal statistics were used to estimate gene flow between wood turtle populations and were compared with results of the BRIDES selection procedure described below.

### BRIDES analysis

**Fig 1:**
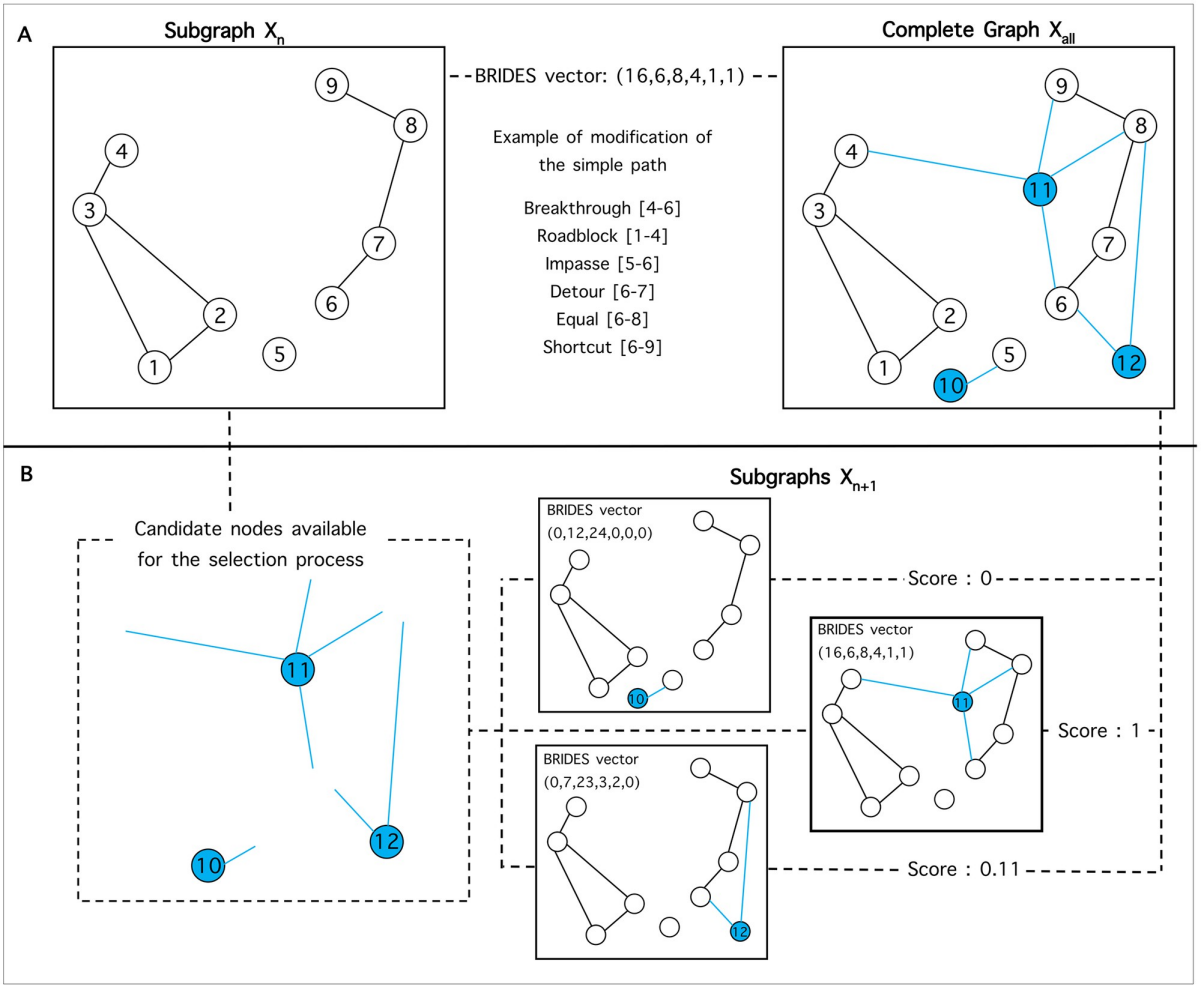
Illustration of the BRIDES algorithm and the selection process. (A) Example of the six types of paths computed in BRIDES by comparing the shortest path between pairs of nodes in subgraph X_n_ (white nodes) and the shortest path between the same pairs of nodes in the complete graph X_all_ including all candidate nodes (blue nodes). In X_all_, the shortest path between any pair of nodes from X_n_ must include at least one of these candidate nodes. (B) Each of the three nodes available for the selection process was iteratively added to X_n_ to create the subgraphs X_n+1_ and corresponding scores were computed with respect to the complete graph X_all_. The selection process selected node 11 as the best candidate (score = 1) to maximize network connectivity while minimizing the number of additional nodes. The weighted model used for the selection process was (1, 0, 0, 0, 1, 1).

Network connectivity was explored using BRIDES v1.2 [57], a software that allows the characterization of dynamic networks through the comparison of shortest paths among pairs of nodes of a subgraph with their respective paths among the same nodes in a complete graph (Fig 1A). Both the complete graph and the subgraph are defined by the users and selected as input networks for the BRIDES algorithm. When applied to population genetic data, the paths identified by BRIDES may thus represent actual gene flow between populations, or past gene flow between remnant populations or metapopulations relative to neutral genetic markers.

Precisely, BRIDES is a polynomial-time algorithm used to characterize different types of paths between a subgraph X_n_ and a complete graph X_all_, where all nodes in X_n_ are a subset of the nodes in X_all_. The shortest paths between pairs of nodes in X_n_ are first computed. Then, the lengths of the paths among the same pairs of nodes are recomputed in X_all_ by forcing these paths to include at least one node present in the complete graph X_all_ but absent in the subgraph X_n_. The comparison of these corresponding path lengths thus allows six types of paths to be distinguished (Fig 1A): a breakthrough (B) is a path that is impossible in X_n_ but possible in X_all_ (path 4–6); a roadblock (R) is a path that is possible in X_n_ but impossible in X_all_ (path 1–4); an impasse (I) is a path that is impossible in both X_n_ and X_all_ (path 5–6); a detour (D) is a path that is shorter in X_n_ than in X_all_ (path 6–7); an equal (E) path has the same length in X_n_ and X_all_ (path 6–8); a shortcut (S) is a path that is longer in X_n_ than in X_all_ (path 6–9). The results are summarized in a vector [B, R, I, D, E, S] containing the numbers of each type of paths estimated by the algorithm (see Fig 1A).

The six different types of paths are meant to capture specific network properties when forcing subgraph paths to pass through additional nodes. With respect to conservation genetic data, shortcuts are indicative of the addition of new populations that will in turn improve gene flow, whereas detours are associated with populations that will reduce gene flow. Breakthroughs are created by populations connecting two (or more) components of the network, similar to cut nodes that represent stepping-stones among isolated populations. Equal paths are corresponding to redundant connections among populations for dispersal and gene flow. In contrast, impasses and roadblocks are detrimental to network connectivity by separating populations in isolated components. These latter types of paths are to be avoided in the prioritization process.

### BRIDES selection algorithm

We implemented an iterative selection algorithm in BRIDES to determine which candidate node should be added to X_n_ to maximize the connectivity of X_n+1_ (Fig 1B). Given that each type of path may have a different impact on the choice of candidate nodes, distinct weighted models could be defined by the user for the selection algorithm. For example, one may consider only breakthroughs, equals and shortcuts to maximize network connectivity. In that case, assuming a weight of 1 for each of the selected types of paths, the corresponding model would be (1, 0, 0, 0, 1, 1), also denoted as B^1^E^1^S^1^. At each step of the algorithm, a selection score is then computed as the sum of weighted numbers of path types in the BRIDES vector. The maximum score that can be achieved is estimated by including all candidate nodes in the subgraph X_n_. For comparison purposes, this maximal score is set to one, and suboptimal solutions are scaled by dividing their scores by the maximum value (see example in Fig 1B).

### Weighted models and conservation scenarios

In the present study, five distinct weighting schemes were used for the six types of paths (B, R, I, D, E, S) to select optimal solutions (maximum scores) under different scenarios, with positive weights (denoted with superscripts) attributed to preferred types of paths, negative weights (denoted with subscripts) attributed to rejected types of paths, and null weights (zero) attributed to types of paths ignored in the final score computation. The first weighted model, S^1^ = (0, 0, 0, 0, 0, 1), only considered shortcuts, whereas the second model, B^3^S^1^ = (3, 0, 0, 0, 0, 1), and third model, B^1^S^3^ = (1, 0, 0, 0, 0, 3), also accounted for shortcuts and breakthroughs, but with distinct weights. The fourth model, B^1^R_1_D_1_S = (1, -1, 0, -1, 0, 1), considered the negative impact of roadblocks and detours with respect to breakthroughs and shortcuts. For the fifth model, B^3^D^1^E^2^S^3^ = (3, 0, 0, 1, 2, 3), we used an ecological interpretation of BRIDES in which breakthroughs, detours, equals and shortcuts were all considered to increase the connectivity of the network, but with different weights.

In addition to the five weighting schemes, six scenarios encompassing three different parameters important for management purposes were considered based on their frequent use in conservation studies: genetic diversity (H_E_), estimated population size, and persistence probability (EO ranks). These scenarios were used to generate different subgraphs X_n_ onto which candidate nodes were added in turn to construct subgraphs X_n+1_. For each parameter, pairs of networks (subgraphs) were generated by selecting nodes with either the largest or smallest values, representing nodes that prioritize a proactive (P) or a reactive (R) conservation perspective, respectively. For the first pair of networks, populations with higher (Scenario A_P_) and lower (Scenario A_R_) heterozygosity values (H_E_) were selected as possible subgraphs for the prioritization process. For the next pair of networks, nodes with higher (Scenario B_P_) and lower (Scenario B_R_) estimates of population sizes were selected. Finally, EO ranks were used to select nodes for the fifth (Scenario C_P_) and sixth (Scenario C_R_) networks based on their persistence probability for a period of time. The five weighting models were then applied to the six scenarios to examine their impact on the node selection process.

## Results

### Population graph

**Fig 2:**
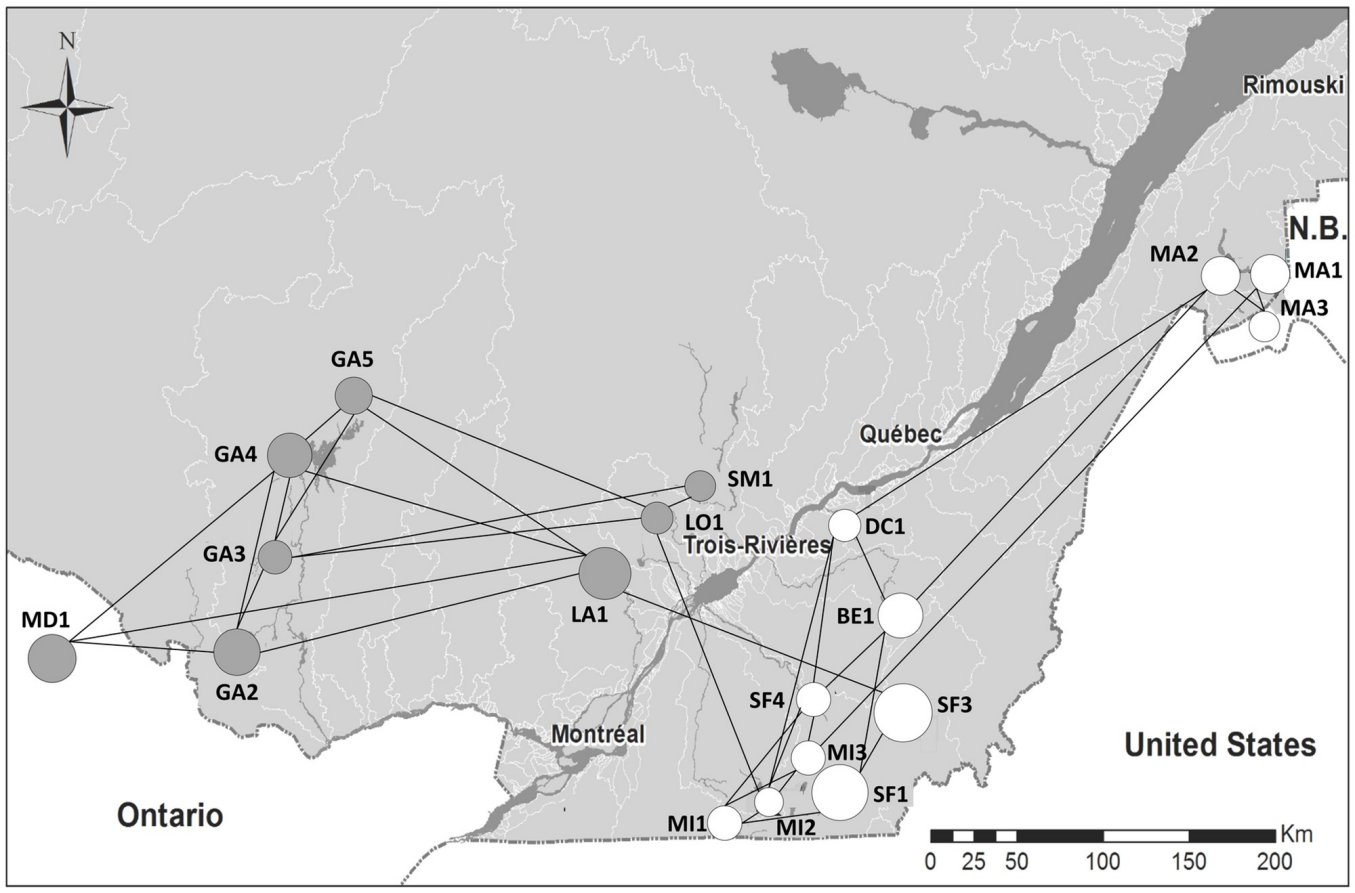
Population graph of 19 wood turtle populations based on conditional genetic distance (cGD). Redundant edges were pruned when they did not contribute to the overall genetic covariance structure. Node sizes are scaled according to the average inverse edge weight (AIEW), where greater AIEW values are reflected through proportinaly larger circular diameters. Populations located on the northern and southern shores of the St. Lawrence River are identified with gray and white nodes, respectively. Source: Esri. " Light Gray Canvas Map" [basemap]. https://www.arcgis.com/home/webmap/viewer.html?layers=ed712cb1db3e4bae9e85329040fb9a49 Orignal data are from Bouchard and colleagues [56].

The wood turtle population graph constructed with cGD resulted in 19 nodes and 37 edges (19% of the edges in a saturated network) after the pruning procedure. Eight nodes were located on the north shore and eleven nodes on the south shore of the St. Lawrence River (Fig 2). Sample sizes and estimated population sizes of different sites ranged respectively from 6 to 56 accounting for 6 to 2000 individuals per node (Table 1).

**Table 1:** Sampled population parameters and corresponding node-based metrics, node removal statistics and BRIDES results.

Shore	Id	N	H_E_	Psize	EO	BC	DC	EC	Strength	AIEW	CC	APL	Selection
**North**	**GA2**	12	0.65	75	BC	3.483	4	**0.85**	82.002	0.049	**0.67**	2.673	0.21
	**GA3**	31	0.72	2000	A	18.3	5	0.55	74.951	0.068	0.4	2.778	0.22
	**GA4**	7	0.63	250	B	5.383	5	**0.94**	**98.256**	0.051	0.6	2.68	0.00
	**GA5**	15	0.62	2000	AB	12.22	4	0.58	64.178	0.064	0.5	2.745	0.07
	**LA1**	11	0.701	50	BC	25.05	5	**1**	**114.731**	0.044	0.4	2.948	0.43
	**LO1**	21	0.754	50	B	**47.18**	4	0.24	56.732	0.071	0.33	**3.281**	0.50
	**MD1**	18	0.724	100	B	0	3	0.76	64.692	0.047	**1**	2.627	0.00
	**SM1**	56	0.771	250	AB	0	2	0.13	26.862	**0.074**	**1**	2.68	0.00
**South**	**BE1**	24	0.634	100	B	22.6	5	0.09	**96.79**	0.053	0.2	2.843	**1.00**
	**DC1**	17	0.736	50	AB	13.23	4	0.05	55.792	0.073	0.5	2.765	0.42
	**MA1**	9	0.68	20	BC	9.4	3	0.04	51.131	0.06	0	2.706	**0.93**
	**MA2**	10	0.625	30	AB	7.5	3	0.03	46.923	0.065	0.33	2.68	0.31
	**MA3**	25	0.75	25	AB	0.7	2	0.01	26.783	**0.075**	0	2.595	0.17
	**MI1**	20	0.73	500	AB	8.45	4	0.08	60.582	0.07	0.5	2.778	0.08
	**MI2**	20	0.71	30	BC	**49.63**	5	0.08	64.001	**0.079**	0.4	**3.353**	0.78
	**MI3**	6	0.74	100	B	12	4	0.05	59.394	0.068	0.5	2.77	0.23
	**SF1**	6	0.74	25	AB	**27.82**	3	0.17	72.665	0.041	0	**2.987**	**1.00**
	**SF3**	6	0.61	6	C	23.82	2	0.38	50.147	0.04	0	2.889	0.36
	**SF4**	13	0.73	25	BC	5.233	5	0.06	73.463	0.069	0.5	2.732	0.62

Site identification (Id), number of genotyped individuals (N), expected heterozygosity (H_E_), estimated population size (PSize), element occurrence rank (EO), betweenness centrality (BC), degree centrality (DC), eigenvector centrality (EC), strength, average inverse edge weight (AIEW), clustering coefficient (CC), average path length (APL) estimated after node removal, and selection probability of each node using the BRIDES’s stepwise selection procedure (Selection). Values in bold indicate the top three populations within each of the metrics, node removal and the selection process.

### Network metrics and node removal

The population graph was analyzed with different node-based metrics to determine which sites contributed most to network connectivity (Table 1). One node (GA3) ranked top in all metrics, whereas two nodes ranked at the very bottom (MA1 and MA2). Above all, these results illustrated a clear separation between sites located on the northern and southern shores of the St. Lawrence River, and the importance of only two edges connecting them. Several nodes with high betweenness centrality values may act as stepping-stones or bridges [38]. Namely, four of the nodes with large betweenness values (MI2, LO1, LA1, SF3) connected the two shores together. In contrast, nodes with a high-clustering coefficient may act as an anchor of interconnected groups of nodes. The maximum value of the clustering coefficient was obtained for two nodes on the north shore (SM1 and MD1), whereas four nodes on the south shore had null values. To further identify nodes that contributed to both direct (immediate connection) and indirect (connection to its neighbors) network connectivity, node eigenvector centrality was computed. Once again, our results show opposite patterns for nodes located on distinct shores, with those on the northern shore exhibiting much larger eigenvector centrality values compared to nodes in the south. Finally, AIEW was calculated to provide estimates of the numbers of migrants to and from a node. Nodes with higher values (AIEW > 0.065) were all located on the south shore of the St. Lawrence River (Table 1). Average path length of the complete network was 2.708. While the removal of a single node (MI2) increased the value up to 3.353 (Table 1), the removal of six individual nodes (MA3, MD1, GA2, GA4, MA24, SM1) provided results close to that of the complete graph (ranging between 2.595 and 2.706), meaning less contributions to network connectivity. There were no cut nodes in our population graph.

### BRIDES selection procedure

**Fig 3:**
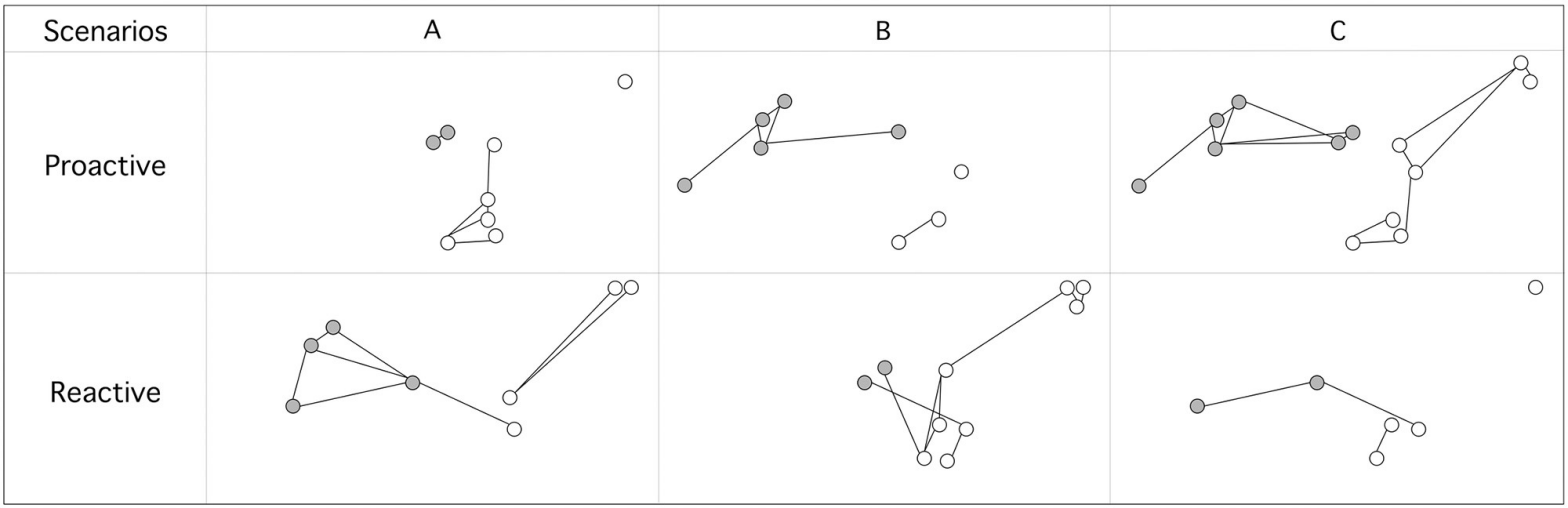
Different population subgraphs based on distinct node selection parameters using a proactive or reactive conservation approach. A: Node selection based on heterozygosity (H_E_) values. B: Node selection based on estimated population size. C: Node selection based on element occurrence (EO) values. For each of these experimental scenarios, nodes included in the subgraph represented populations with the largest (Proactive) or smallest (Reactive) parameter values. Populations located on the northern and southern shores of the St. Lawrence River are identified with gray and white nodes, respectively.

Using the BRIDES selection process, we explored which nodes were the most important for network connectivity using six subgraphs that were constructed from the complete population graph including 19 wood turtle populations under different conservation scenarios. Depending on the criteria selected, these networks contained between 6 and 13 nodes (Fig 3). Networks based on genetic heterozygosity included populations with H_E_ values greater than (Scenario A_P_) or lower than 0.72 (Scenario A_R_). Those based on estimated population sizes were created by including populations with more (Scenario B_P_) or less than (Scenario B_R_) 75 individuals. Networks based on element occurrence included populations with EO ranks greater than BC (Scenario C_P_) or lower than B (Scenario C_R_) (Table 1). The five weighting models were then used to examine their impact on the node selection procedure and explore BRIDES flexibility under a wide range of experimental conditions. The identity of the selected nodes was very similar (90%) regardless of the weighted model selected for a given scenario (S1 Fig and S1 Table).

**Fig 4:**
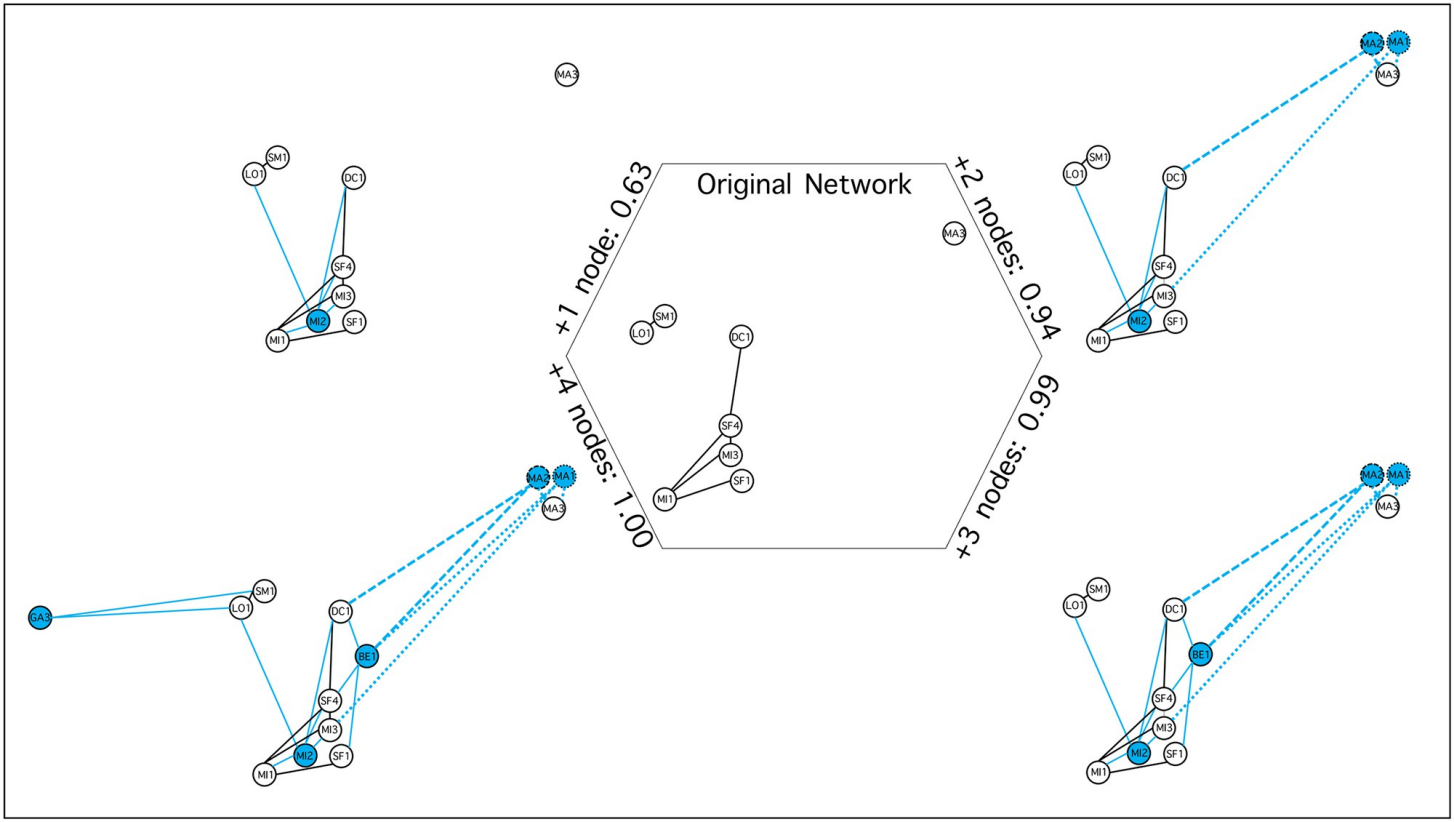
BRIDES node selection process for scenario Ap and Model B^3^D^1^E^2^S^3^ (3, 0, 0, 1, 2, 3). All selection steps are shown, even when multiple solutions had identical scores. In such cases, distinct solutions (i.e., MA1 or MA2) are identified by dashed edges connecting the selected nodes. Maximum score (1.00) was reached with the addition of four candidate nodes. At each step of the procedure, the selected nodes and their corresponding links are presented in blue.

Results of the node selection process revealed that regardless of the weighting schemes and conservation scenarios, maximum scores were obtained by adding from one to six nodes to the subgraph X_n_ (Table 2). A positive relationship was observed between the number of candidate nodes required to reach the maximum score and the complexity of the weighting scheme, with higher complexity models allowing for more diverse path types, but also requiring more candidate nodes (Kendall Tau = 0.634, p < 0.001) (see S1 Fig for examples). Namely, for the first weighting scheme (Model S^1^) that only accounted for shortcuts (complexity of 1), three out of six scenarios returned no solution as no shortcuts were possible by adding candidate nodes to the subgraph. In three other cases, one or two candidate nodes were required to reach a maximal score. Models B^3^S^1^ and B^1^S^3^ considered shortcuts as well as breakthroughs (complexity of 2), but with different weighting schemes, and the number of nodes required to reach the maximum score varied from one to three. Model B^1^R_1_D_1_S^1^ contained contrasting roadblocks and detours with respect to breakthroughs and shortcuts (complexity of 4), and two to five candidate nodes were required to reach a maximal score. Finally, Model B^3^D^1^E^2^S^3^ accounted for breakthroughs, detours, equals, and shortcuts (complexity of 4), and three to six nodes were required to reach a maximal score. In some cases, multiple solutions with equal scores were available at any given step of the procedure. To represent these equally multiple solutions, we depicted alternative paths with different types of edges in corresponding graphs (Fig 4). This redundancy was observed in the results from five of the six subgraph X_n_ selections for at least one step of the node selection procedure (Table 2).

**Table 2:** Summary of the BRIDES node selection procedure providing the minimum number of nodes required to reach a maximal score, and the number of multiple solutions with the same score for different scenarios and weighted model schemes.

**Scenarios**	**A**_**P**_					**A**_**R**_				
**Models**	S^1^	B^3^S^1^	B^1^S^3^	B^1^R_1_D_1_S^1^	B^3^D^1^E^2^S^3^	S^1^	B^3^S^1^	B^1^S^3^	B^1^R_1_D_1_S^1^	B^3^D^1^E^2^S^3^
**No. of nodes**	1	3	3	3	4	-	1	1	3	6
**No. of solutions**	1	2	2	2	2	-	1	1	1	4
**Scenarios**	**B**_**P**_					**B**_**R**_				
**Models**	S^1^	B^3^S^1^	B^1^S^3^	B^1^R_1_D_1_S^1^	B^3^D^1^E^2^S^3^	S^1^	B^3^S^1^	B^1^S^3^	B^1^R_1_D_1_S^1^	B^3^D^1^E^2^S^3^
**No. of nodes**	-	3	3	5	5	1	1	1	2	3
**No. of solutions**	-	5	5	5	4	1	1	1	1	1
**Scenarios**	**C**_**P**_					**C**_**R**_				
**Models**	S^1^	B^3^S^1^	B^1^S^3^	B^1^R_1_D_1_S^1^	B^3^D^1^E^2^S^3^	S^1^	B^3^S^1^	B^1^S^3^	B^1^R_1_D_1_S^1^	B^3^D^1^E^2^S^3^
**No. of nodes**	2	2	2	5	5	-	2	2	2	4
**No. of solutions**	2	1	1	2	2	-	1	1	1	1

A: Node selection based on heterozygosity (H_E_) values. B: Node selection based on estimated population size. C: Node selection based on element occurrence (EO) values. For each scenario, nodes included in the subgraph represented populations with the largest (Proactive) or smallest (Reactive) parameter values. Scenarios for which no nodes were selected (-) are also shown.

## Discussion

### Properties of the BRIDES selection procedure

BRIDES can repetitively assess the impact of multiple conservation scenarios without considering a priori models of population dynamics. The selection process thus allows the user to test different scenarios and weighting schemes to decide which one is best for population prioritization. When applied jointly with PopGraph, BRIDES will select the minimum number of nodes required to optimize gene flow and network connectivity. Moreover, BRIDES can identify multiple solutions with identical scores, thus allowing for alternative node combinations in the prioritization process. Its node selection process is robust for a given subgraph. In addition, BRIDES is highly flexible and provides the user with the opportunity to determine which types of paths need to be accounted for, and how they should be weighted to compute scores.

The BRIDES selection algorithm ranks all nodes based on their contribution to path lengths in the subgraph X_n_ with respect to the complete network X_all_ (Fig 1). Based on hypothetical conservation scenarios, our results highlighted the relative importance of some candidate nodes in the complete network, when their addition increased the numbers of shortcuts or breakthroughs. However, node selection can vary depending on the nodes chosen for the construction of subgraph X_n_. When divergent scenarios are considered, BRIDES provides an objective criterion to guide the decision-making process. Yet, considering the high variation observed in the ranking given the subgraph used, the prioritization process should account for sensitivity to missing data and subgraphs must be chosen carefully. Namely, we suggest to experiment with different measures of interest for management purposes. Our results also highlight the need to further evaluate potential sources of uncertainty in network analysis, especially when applied to conservation biology where the impact of missing nodes on path lengths and component connectivity has yet to be assessed [65, 66].

Important ranking information is largely provided by node-based metrics, as nodes can be compared across a multitude of indices operating at different scales and patterns of connectivity. Cross and colleagues [45] argue that this flexibility can be beneficial when top-ranking nodes are first targeted by conservation actions. Yet, as pointed out by Creech and colleagues [29], the choice of a biologically relevant metric to quantify connectivity has a direct effect on the prioritization rankings, and our results revealed such inconsistencies among node-based metrics (Table 1). Similarly, node removal is highly sensitive, and it may produce inconsistent outcomes in habitat connectivity analysis [43, 44]. In agreement with previous studies, we trust that network metrics are valuable tools to understand complex patterns of connectivity and gene flow. On the other hand, we believe that node-based metrics should not be applied as the only prioritization criteria. Contrary to centrality measures focusing on static networks, BRIDES allows the analysis of dynamic networks onto which candidate nodes and edges are added. As shown in our results, the nodes more frequently selected by BRIDES were not always consistent with those selected by node-based metrics and node removal (Table 1). BRIDES identifies six different types of paths, each one capturing specific properties of path lengths within evolving networks. Yet, as it targets nodes based on their connectivity, BRIDES might miss some important peripheral or isolated nodes. For example, isolated nodes in a subgraph may remain disconnected when their selection leads to more detours and roadblocks than the selection of a central node (see S1 Fig, Scenario C_R_). For that reason, BRIDES may be used jointly with complementary tools, such as phylogenetic networks developed by Volkman and colleagues [42] to account for genetic distinctiveness and identify peripheral populations.

### Prioritization process of wood turtle populations

In the context of wood turtle conservation genetics, the results we obtained with population graphs are consistent with previous landscape genetics analyses [56], however, relationships among populations are much more detailed. As opposed to pairwise methods, population graphs account for genetic covariance among all populations simultaneously [37, 67]. It is already known that the St. Lawrence River acts as a barrier to gene flow among wood turtle populations located on opposite shores [51, 56, 68] but the population graph revealed which populations and corresponding links are more connected across the river (Fig 2). The network also depicted a detailed account of the interaction patterns between populations located on the two shores. We observed four nodes (LA1, LO1, MI2, SF3) linking the two shores, which could be viewed as stepping-stones or bridges between populations [38]. The results obtained after node removal also support the interconnection among populations on the north shore, except for two nodes linking the southern shore (LA1, LO1) that affect average path lengths. This implies that gene flow among north shore populations makes them less susceptible to local extinctions. On the other hand, populations on the south shore are more loosely connected, except for some sites exhibiting high values of AIEW (MA3, MI2). Metapopulation dynamics should be further investigated for these sites since AIEW is an accurate measure of reproductive success and the number of migrants between nodes [63].

The BRIDES algorithm was applied in this study to demonstrate how it may be used to select wood turtle populations with either a reactive or proactive prioritization procedure. To do so, we considered six hypothetical scenarios and five different weighting schemes, and applied a node selection procedure to prioritize populations. For example, the first scenario only included populations with large heterozygosity values. Even when neutral genetic diversity is not correlated to functional diversity [69], heterozygosity may still identify well-connected sites, large population sizes despite isolation, habitat fragmentation, and perturbance. In a proactive conservation plan, such populations with low vulnerability may be selected first to protect the genetic variability of the species before it erodes [15–17]. Namely, the link between the north and south shores of the St. Lawrence River was included in the subgraphs only when a proactive conservation perspective was used. meaning that some of theses populations might be characterized by small H_E_, population sizes, and high EO ranks.

Regardless of their genetic diversity, the topological relationships among nodes within the network may be of importance for connectivity [70]. Peripheral populations associated with MA sites are thus of great interest to quantifying functional connectivity. Although MA3 presents high neutral genetic diversity, all MA sites are clustered together in Bouchard and colleagues [56]. Likewise, node-based metrics for these populations were in the low to mid-range, except for MA3, which exhibited a large AIEW value. Removal of these three nodes had no impact of the average path length of the network. These results are in stark contrast with those obtained with BRIDES, where the MA1 was very likely to be selected to maximize network connectivity. Such a discrepancy illustrates that node-based metrics may overlook important nodal features that BRIDES actually considers during its selection process.

In Québec, riverbeds and riverbanks which are part of wood turtle habitats are protected in public land, but stronger management actions are still needed to ensure long-term survival of populations [71]. To do so, one can apply BRIDES to prioritize populations in a reactive fashion. That is that populations with the smallest sizes–those needing high priority management actions such as nest protection from predators or a head-starting program–would first be selected to construct a subgraph (Scenario B_R_, Fig 3 and S1 Fig). As the resulting subgraph may be disconnected and these small populations may face extirpation, one could also adopt a proactive perspective to protect larger populations that support them. More precisely, large turtle populations, as a source of genetically similar individuals, may add redundancy and resilience to population extirpation in the network. Namely, BRIDES can be applied to select these candidate populations based on path lengths and other connectivity indices (Model B^3^D^1^E^2^S^3^, S1 Fig). In this particular case, populations BE1, MI3 and GA5 (Model B^3^D^1^E^2^S^3^, S1 Fig) would be selected by BRIDES to guide the complex decision-making process of population prioritization.

Subgraph constructions in our study were based on proactive and reactive procedures to illustrate the method, but it is entirely up to the user to select populations in the starting subgraphs. The subgraphs could also be based on characteristics independent of the species, such as land ownership or jurisdictions involved allowing for nesting sites or for hibernacula that can be easily protected and managed. Because the choice of populations in subgraphs greatly influences the subsequent selection of candidate nodes, we suggest to use practical conservation criteria to select the nodes of a subgraph, especially when BRIDES is applied for population prioritization.

Uncertainties about genetic connectivity, genetic differentiation, isolation by distance, and population assignment may all be addressed with a single heuristic approach: the network. Moreover, many levels of connectivity can be considered at a different time and spatial scales when using evolving networks, especially those analyzed with BRIDES. Network analysis also offers an alternative model to population genetic analysis when populations are not considered to be at a mutation-drift balance and depart from the Hardy-Weinberg equilibrium [70, 72, 73]. Yet, networks are not free of a priori assumptions that are associated with the choice of a genetic distance, the pruning procedure, and the criteria selected for the construction of the subgraph. H_E_, notably, may not be the most relevant criteria since it was very similar in several populations. In the present case, subgraphs were created by removing nodes and their corresponding edges from the complete population graph among 19 wood turtle populations. Considering that the graph pruning method is based on the removal of redundant edges that do not contribute to the overall genetic covariance of the network, population graphs should be re-estimated every time a node is removed.

Given the specific, historic life traits of long-lived organisms with low recruitment and late sexual maturity, the genetic characterization of recent perturbation events can be challenging [74]. As network topology stabilizes rapidly following a perturbation event, network analysis could be part of the solution to detect more precisely the genetic signal [67, 75]. For example, many species with high levels of gene flow could benefit from this network approach, like many turtle and tortoise species [74, 76]. Likewise, it could apply to other long-lived species of vertebrates [77, 78], as well as some plant species without any geographical barriers to gene flow [79, 80]. More precisely, BRIDES represents an interesting avenue to study trophic networks or to compare various scenarios during the design of protected area networks. Managers could benefit from using such tools to understand the impact of the addition or removal of a species within a food web, whether it be through the impact of the introduction of an invasive exotic species or the selection of a biocontrol agent. Namely, a scenario with fewer breakthroughs or shortcuts could be preferred to limit the impact of introduced invasive species. BRIDES could also be applied to optimize the connectivity among isolated habitat patches, with respect to historical levels of connectivity, when protected areas are designed. In a time where environmental challenges are pressing and greater emphasis is given to temporal scales when modeling climate change, population graphs and evolving networks may provide a better understanding of the evolving ecosystem, trophic networks and population structures.

## Supporting information

S1 Fig Results of the BRIDES node selection procedure for two scenarios and three weighted models. In each case, networks with optimal scores while minimizing the number of candidate nodes are presented. Nodes from the subgraph X_n_ are depicted by empty circles, whereas candidate nodes and corresponding edges in X_n+1_ are depicted by blue circles.
(TIF)

S1 Table Results of the BRIDES node selection procedure for six scenarios (A, B, C) and five weighted models (S^1^, B^3^S^1^, B^1^S^3^, B^1^R_1_D_1_S^1^, B^3^D^1^E^2^S^3^). For each node, the initial presence of a population in the subgraph is identified by (x) and its selection (1) or not (0) in at least one of the optimal solutions is indicated. Scenarios for which no nodes were selected (-) are also shown.
(PDF)
