# Supplementary figures and images for: Applying novel connectivity networks to wood turtle populations to provide comprehensive conservation management strategies for species at risk
Source: PLoS One. 2022 Aug 12;17(8):e0271797. doi: 10.1371/journal.pone.0271797 (PMC9374220; doi:10.1371/journal.pone.0271797)

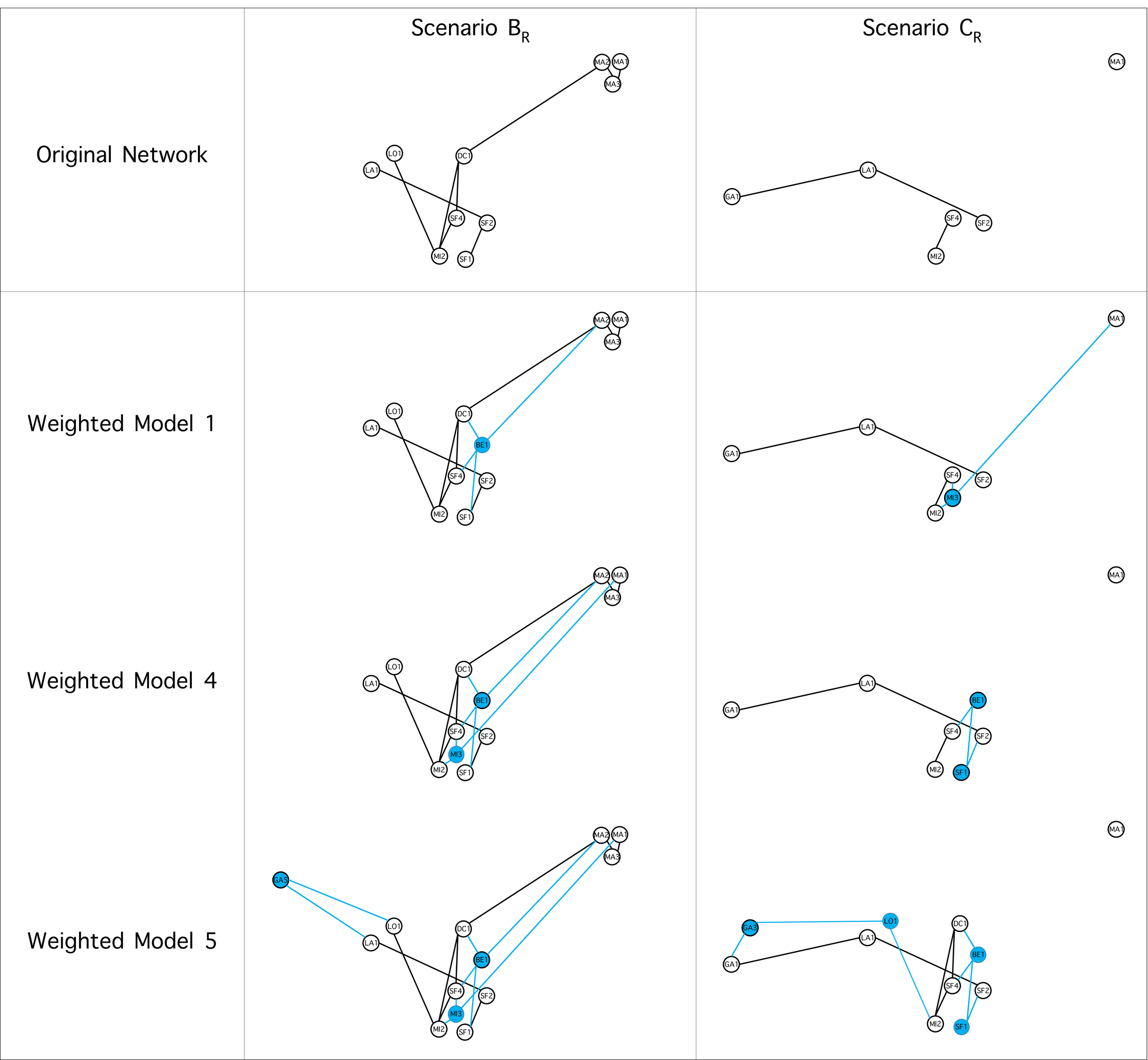

Supplement: S1 Fig — In each case, networks with optimal scores while minimizing the number of candidate nodes are presented. Nodes from the subgraph Xn are depicted by empty circles, whereas candidate nodes and corresponding edges in Xn+1 are depicted by blue circles. (TIF) [file pone.0271797.s001.tif]
