# Supplementary material for: Applying novel connectivity networks to wood turtle populations to provide comprehensive conservation management strategies for species at risk
Source: PLoS One. 2022 Aug 12;17(8):e0271797. doi: 10.1371/journal.pone.0271797 (PMC9374220; doi:10.1371/journal.pone.0271797)
Supplement: S1 Table — For each node, the initial presence of a population in the subgraph is identified by (x) and its selection (1) or not (0) in at least one of the optimal solutions is indicated. Scenarios for which no nodes were selected (-) are also shown. (PDF) [file pone.0271797.s002.pdf]

S1 Table. Results of the BRIDES node selection procedure for six scenarios (A, B, C) and five weighted models (S^1, B^3S^1, B^1S^3, $B^1R_1D_1S^1$, $B^3D^1E^2S^3$).

Scenarios		AP					AR					BP					BR					CP					CR				
Model		S^1	B^3S^1	B^1S^3	$B^1R_1D_1S^1$	$B^3D^1E^2S^3$	S^1	B^3S^1	B^1S^3	$B^1R_1D_1S^1$	$B^3D^1E^2S^3$	S^1	B^3S^1	B^1S^3	$B^1R_1D_1S^1$	$B^3D^1E^2S^3$	S^1	B^3S^1	B^1S^3	$B^1R_1D_1S^1$	$B^3D^1E^2S^3$	S^1	B^3S^1	B^1S^3	$B^1R_1D_1S^1$	$B^3D^1E^2S^3$	S^1	B^3S^1	B^1S^3	$B^1R_1D_1S^1$	$B^3D^1E^2S^3$
**Shore**	**Node Id**																														
**North**	**GA2**	0	0	0	0	0	x	x	x	x	x	-	0	0	1	1	0	0	0	0	0	0	0	0	1	1	x	x	x	x	x
	**GA3**	0	0	0	0	1	-	0	0	1	1	x	x	x	x	x	0	0	0	0	0	x	x	x	x	x	-	0	0	0	1
	**GA4**	0	0	0	0	0	x	x	x	x	x	x	x	x	x	x	0	0	0	0	0	x	x	x	x	x	-	0	0	0	0
	**GA5**	0	0	0	0	0	x	x	x	x	x	x	x	x	x	x	0	0	0	0	1	x	x	x	x	x	-	0	0	0	0
	**LA1**	0	0	0	0	0	x	x	x	x	x	-	1	1	1	1	x	x	x	x	x	0	0	0	1	1	x	x	x	x	x
	**LO1**	x	x	x	x	x	-	0	0	0	1	-	1	1	1	1	x	x	x	x	x	x	x	x	x	x	-	0	0	0	1
	**MD1**	0	0	0	0	0	-	0	0	0	0	x	x	x	x	x	0	0	0	0	0	x	x	x	x	x	-	0	0	0	0
	**SM1**	x	x	x	x	x	-	0	0	0	0	x	x	x	x	x	0	0	0	0	0	x	x	x	x	x	-	0	0	0	0
**South**	**BE1**	1	1	1	1	1	x	x	x	x	x	x	x	x	x	x	1	1	1	1	1	x	x	x	x	x	-	1	1	1	1
	**DC1**	x	x	x	x	x	-	0	0	0	1	-	1	1	1	1	x	x	x	x	x	x	x	x	x	x	-	0	0	0	0
	**MA1**	0	1	1	1	1	x	x	x	x	x	-	1	1	1	1	x	x	x	x	x	1	1	1	1	1	x	x	x	x	x
	**MA2**	0	1	1	1	1	x	x	x	x	x	-	0	0	0	0	x	x	x	x	x	x	x	x	x	x	-	0	0	0	0
	**MA3**	x	x	x	x	x	-	0	0	1	1	-	0	0	0	0	x	x	x	x	x	x	x	x	x	x	-	0	0	0	0
	**MI1**	x	x	x	x	x	-	0	0	0	1	x	x	x	x	x	0	0	0	0	0	x	x	x	x	x	-	0	0	0	0
	**MI2**	0	1	1	1	1	-	0	0	0	1	-	1	1	1	1	x	x	x	x	x	1	1	1	1	1	x	x	x	x	x
	**MI3**	x	x	x	x	x	-	0	0	0	1	x	x	x	x	x	0	0	0	1	1	x	x	x	x	x	-	0	0	0	0
	**SF1**	x	x	x	x	x	-	1	1	1	1	-	1	1	1	1	x	x	x	x	x	x	x	x	x	x	-	1	1	1	1
	**SF3**	0	0	0	0	0	x	x	x	x	x	-	1	1	1	0	x	x	x	x	x	0	0	0	1	1	x	x	x	x	x
	**SF4**	x	x	x	x	x	-	0	0	0	1	-	1	1	1	1	x	x	x	x	x	1	0	0	1	1	x	x	x	x	x

For each node, the initial presence of a population in the subgraph is identified by (x) and its selection (1) or not (0) in at least one of the optimal solutions is indicated. Scenarios for which no nodes were selected (-) are also shown.
